# Vestibularisschwannome: Diagnose – Therapie – Nachsorge

**DOI:** 10.1007/s10354-020-00800-y

**Published:** 2021-01-13

**Authors:** Erdem Yildiz, Valerie Dahm, Christian Matula, Christoph Arnoldner

**Affiliations:** 1 Universitätsklinik für Hals‑, Nasen- und Ohrenkrankheiten, Medizinische Universität Wien, Währinger Gürtel 18–20, 1090 Wien, Österreich; 2 Universitätsklinik für Neurochirurgie, Medizinische Universität Wien, Währinger Gürtel 18–20, 1090 Wien, Österreich

**Keywords:** Schädelbasischirurgie, Otologische Chirurgie, Kleinhirnbrückenwinkel, Schwannombehandlung, Neurinom, Skull base surgery, Otologic surgery, Cerebellopontine angle, Schwannoma treatment, Neurinoma

## Abstract

Vestibularisschwannome können die Lebensqualität von Patienten stark beeinträchtigen. Neben einer eingeschränkten Hörfunktion wird die Gesichtslähmung hierbei als besonders störend empfunden. Unterschiedliche Wachstumsraten dieser gutartigen Tumore erschweren die zeitliche Vorhersage einer funktionellen Beeinträchtigung von Hirnnerven. Deshalb ist ein regelmäßiges Update zu aktuellen Therapiestrategien und alternative Behandlungsmöglichkeiten sowohl für Ärzte als auch Patienten relevant.

## Abstract (en)

Vestibular schwannomas can severely impair the quality of life of patients. Next to impaired hearing function, facial palsy is perceived as particularly disturbing in this context. Varying growth rates of these benign tumors complicate a prediction of functional impairment of cranial nerves. Therefore, a regular update on current therapeutic strategies and alternative treatment options is relevant for both physicians and patients.

Vestibularisschwannome (VS) sind gutartige Tumore (klassifiziert als WHO-Grad I), die in der Regel von den Schwann-Zellen des vestibulären Anteils des Nervus vestibulocochlearis ausgehen. In seltenen Fällen ist sein Ursprung am cochleären Anteil des achten Hirnnerven. Die Inzidenz der sporadischen VS liegt bei ca. 1–2/100.000 im Jahr, dies entspricht etwa 6–7 % aller intrakraniellen Tumore [1]. Bilaterale VS, die im Rahmen einer Neurofibromatose Typ 2 auftreten, sind als separate Entität sowohl biologisch als auch klinisch zu behandeln.

Der offene Zugang zu Magnetresonanztomografien (MRT) sowie die Verbesserung dieser radiologischen Untersuchungsmöglichkeit, zum Beispiel durch die zusätzliche Gabe von Gadolinium-haltigen Kontrastmitteln, ermöglichen heutzutage eine frühere Diagnose. Ein echter Inzidenzanstieg konnte bis heute nicht nachgewiesen werden [2, 3].

## Diagnose & Klinik

**Figure 1:**
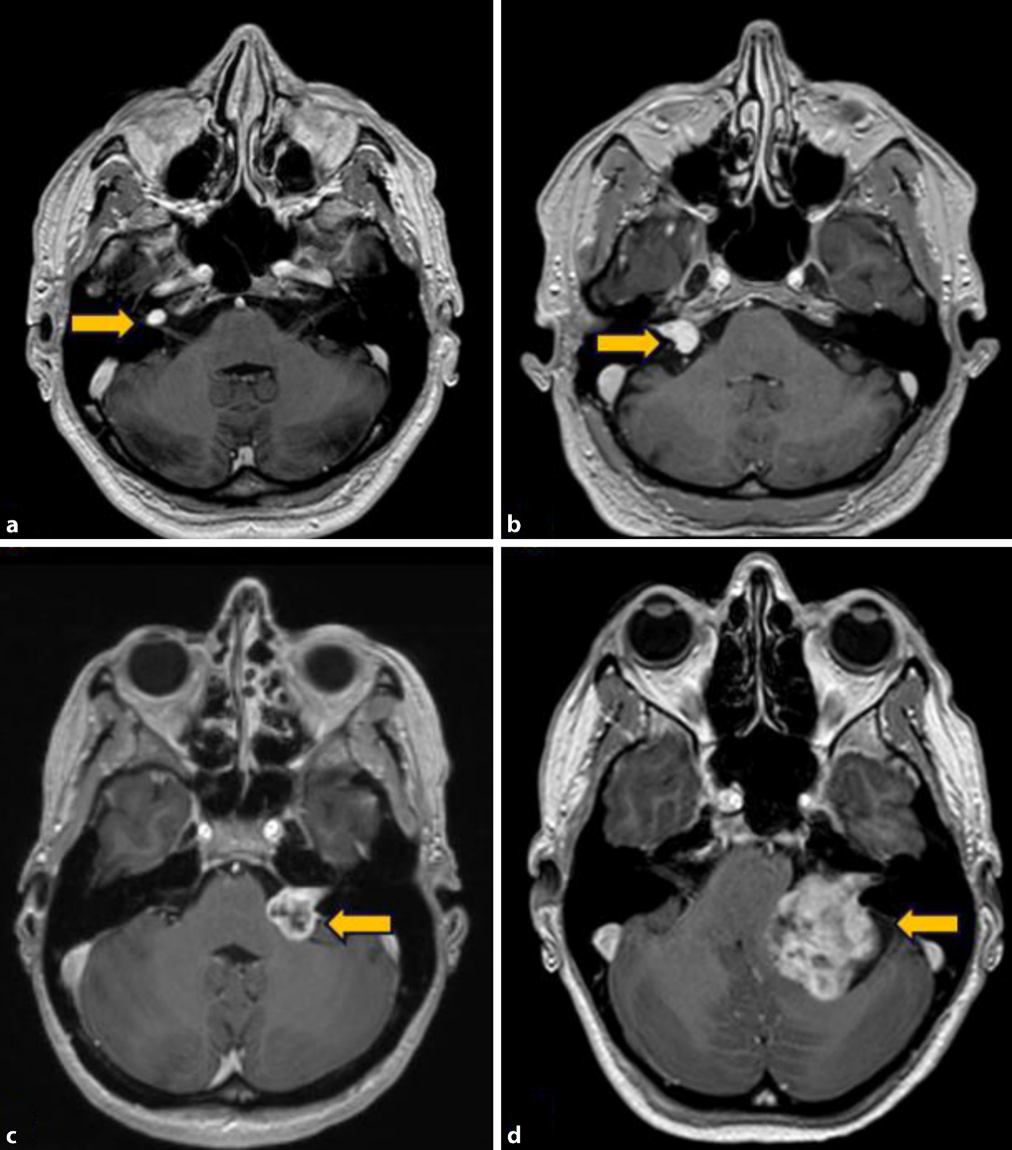

MRT, Ton- und Sprachaudiometrie, Vestibularis-Diagnostik und Erhebung der Fazialisfunktion sind die wesentlichen Untersuchungen zur Abklärung eines VS. Die Bildgebung mittels MRT ist heute die Methode der 1. Wahl für die Diagnose- und Verlaufsbeurteilung, wobei spezielle Sequenzprotokolle angewendet werden. Mithilfe der Bildgebung wird das VS klassifiziert. Ein häufig verwendeter, internationaler Standard zur Tumoreinteilung ist die Koos-Klassifikation, welche die VS nach anatomischer Lage und Tumorgröße graduiert (Grad I–IV, Tab. 1, Abb. 1). Intralabyrinthäre VS (Lage z. B. in Cochlea, Vestibulum, Bogengängen) werden als separate Entität gewertet.

**Table 1:** 

Tumorgrad	Lagebeschreibung des Tumors
Grad I	Intrameataler Tumor
Grad II	Tumorprotrusion Richtung KHBW ^a^ (ohne Kontakt zum Hirnstamm)
Grad III	Tumor besetzt die Cisterna pontocerebellaris (Hirnstamm wird nicht versetzt)
Grad IV	Tumor verschiebt Hirnstamm und Hirnnerven

^a^ Kleinhirnbrückenwinkel

Im frühen Stadium des VS kann eine initiale Schwindelsymptomatik bestehen, die individuell unterschiedlich stark ausgeprägt sein kann. Etwa 10–15 % der Patienten klagen über zunächst unklaren Schwindel ohne Hörminderung und Tinnitus, welcher oft als unsystematische Gangunsicherheit beschrieben wird und in der Dunkelheit zunimmt [1].

Das Leitsymptom von sporadischen VS ist die einseitige Hörminderung (94 %, [5]). Eine einseitige, asymmetrische Hörminderung kann mittels Audiometrie diagnostiziert werden. Rezente Guidelines aus den Vereinigten Staaten empfehlen, dass ab einer interauralen Differenz im Reintonaudiogramm ≥10 dB an zwei oder mehr aneinander folgenden Frequenzen oder ab einer Differenz ≥15 dB einer beliebigen Frequenz eine MRT Indikation besteht. Hierbei würde das Screening einer Asymmetrie von ≥15 dB bei 3 kHz die relative Häufigkeit vorhandener VS im MRT erhöhen [6]. Während VS Patienten im Anfangsstadium nur eine geringe Asymmetrie im Reintonaudiogramm vorweisen, ist das Sprachverstehen des betroffenen Ohrs oft bereits deutlich eingeschränkt [7].

Aufgrund der anatomischen Nähe und des geringen Raums im inneren Gehörgang sind eine Funktionsstörung des Nervus facialis und fokalneurologische Ausfälle möglich. Für die Beurteilung der Gesichtslähmung hat sich die Klassifizierung nach House-Brackmann durchgesetzt (HB°I–VI, Tab. 2).

**Table 2:** 

HB°	Bedeutung	Grobe Beschreibung der Fazialisparese
I	Normal	Normale Funktion im gesamten Innervationsgebiet
II	Gering	Geringe Schwäche bei naher Inspektion/ mögliche minimale Synkinesie
III	Moderat	Dysfunktion sichtbar, allerdings keine entstellende Asymmetrie
IV	Mittelschwer	Offenkundige Schwäche und/oder entstellende Asymmetrie, inkompletter Lidschluss
V	Schwer	Kaum erkennbare Bewegung der Gesichtsmuskulatur
VI	Paralyse	Keine Bewegung der betroffenen Gesichtsmuskulatur

*HB* House Brackmann

## Therapieoptionen

Jedes VS erfordert ein individuell angepasstes Therapiekonzept und bedarf einer interdisziplinären Beurteilung. Prinzipiell kommen als Therapieoptionen Observanz (Wait & Scan), Radiotherapie und die chirurgische Resektion in Frage.

Die Entscheidung der jeweiligen Therapie ist von vielen Faktoren wie Wachstumsgeschwindigkeit, Tumorgröße und -lage, Hörvermögen des Patienten, Alter, Vorerkrankungen, Vertigo und nicht zuletzt von der individuellen Entscheidung des Patienten abhängig.

## Wait & Scan – Wie lange Observanz?

Wait & Scan bedeutet, dass im Abstand von zunächst 6 Monaten (danach jährlich) eine neuerliche MRT (und Audiometrie mit Sprachverstehen) durchgeführt wird. Bei dieser Strategie kann erhoben werden, ob das VS wächst und wie schnell die Wachstumsdynamik ist. In der Literatur werden unterschiedliche Wachstumsraten von VS berichtet. Eine kürzlich erschienene Studie beschreibt volumetrische Wachstumsraten von VS in einem Zeitfenster von 6 Jahren, wobei 66 % der VS wuchsen, etwa 30 % davon belegten eine Wachstumsrate von 100 % pro Jahr (sogenannte „fast growing tumors“, [9]). Tendenziell schnell wachsende Tumore (>2 mm/Jahr) sollten chirurgisch reseziert werden. Dieses Vorgehen ist vor allem bei Erstdiagnose von kleinen Tumoren sinnvoll. Tumore die kontinuierlich wachsen (wenn auch nur 1–2 mm pro Jahr) sollten unbedingt therapiert werden bevor größere Tumorstadien (Koos°III–IV) vorliegen.

## Strahlentherapie

Die konventionelle Strahlentherapie mit Linearbeschleunigern wurde früher als Therapieoption für VS im Wiener AKH angewandt. Heute ist die moderne stereotaktische Radiotherapie mittels Gamma Knife als präzise Hochdosistherapie mit einem schnellen Abfall der Bestrahlungsintensität etabliert, die lediglich eine singuläre Behandlung erfordert. Vorteile dieser seit 1992 im Wiener AKH angewandten Therapie sind der zeitsparende und unkomplizierte Ablauf und eine gute lokale Tumorkontrollrate von ca. 92 % [10]. Diese Zahl konnte in einer rezent durchgeführten Meta-Analyse von stereotaktischen bestrahlten zystischen VS, einem schnellwachsenden Subtyp, bestätigt werden [11]. In einer Studie der Medizinischen Universität Wien wurde das Gehör von VS vor und nach Gamma Knife Therapie evaluiert. Hier konnte gezeigt werden, dass bei lediglich 55 % der Patienten mit gutem Gehör, vor der Gamma Knife Therapie dieses auch 2 Jahre nachher erhalten war. Im Langzeit Follow-up von 10 Jahren waren es nur mehr 34 % der Patienten [10].

## Chirurgische Therapie

Zu Beginn der mikrochirurgischen Ära war die komplette chirurgische Resektion Priorität – häufig auch zu Lasten der Nervenfunktion. Durch die Verbesserung der chirurgischen Möglichkeiten wird jedoch ein Fokus auf den Nerven- und Funktionserhalt gelegt ohne auf eine komplette Tumorresektion zu verzichten. Für diese Zielsetzung ist heutzutage das intraoperative Neuromonitoring unabdingbar. Mithilfe eines konstant laufenden Elektromyogramms (EMG) können intraoperativ auftretende Spontanaktivitäten des Gesichtsnervs, durch dessen Manipulation, umgehend an den Chirurgen gemeldet werden. Neben der jahrzehntelangen Expertise im Wiener AKH half das Neuromonitoring einen Funktionserhalt des Gesichtsnervs in über 81 % (translabyrinthärer Zugang), 86 % (subtemporaler Zugang) bzw. 68 % (retrosigmoidaler Zugang, inklusive KOOS°I–IV) der Fälle zwischen 2015 und 2018 zu erreichen. Diese Ergebnisse decken sich mit den Daten einer Meta-Analyse im Umfang von 11.873 Patienten [12].

**Figure 2:**
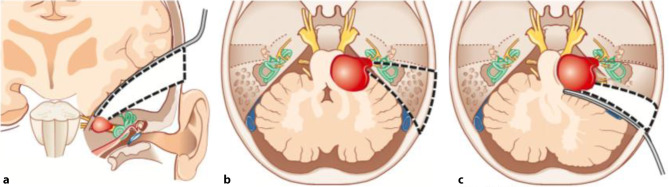

Studien zeigten, dass bei einer annähernd kompletten Resektion („near total resection“, <2 % Residualtumor) selten ein neuerliches Wachstum (0,0–3,5 %, [13]) zu erwarten ist. Jedoch ist bei einer subtotalen Resektion, welche als Entfernung von <95 bis <98 % der Tumormasse definiert wird, in 18,4 bis 73,9 % mit einem neuerlichen Tumorwachstum zu rechnen [14]. Chirurgisch kommen drei verschiedene Zugangswege in Frage (Abb. 2; [15]). Welcher Zugangsweg für einen bestimmten Patienten sinnvoll ist, hängt von der Lage und Ausdehnung des Tumors, dem Hörvermögen und dem Wunsch des Patienten ab.

*Retrosigmoidale (retromastoidale)* Zugänge sind vor allem für Tumore mit großem extrameatalen Anteil geeignet [1]. Hierbei präpariert der Chirurg den Tumor unter einer weitreichenden Exposition der hinteren Schädelgrube. Allerdings sind VS, die weit in den lateralen Anteil des inneren Gehörgangs (Fundus) reichen, oft nicht ausreichend erreichbar und somit nicht komplett hörerhaltend resezierbar.

*Subtemporale Zugänge („Middle Fossa“)* sind vor allem für Patienten mit intrameatalen und fundusnahen VS, welche nur eine geringe Ausbreitung außerhalb des inneren Gehörgangs besitzen, sinnvoll [1].

Prozentuelle Angaben zum Hörerhalt beider Zugänge scheinen abhängig von der Tumorgröße zu sein. Die Therapie intrameataler VS hat eine bessere Hörerhaltungsrate als extrameatale Tumore [16]. Während die Therapie größerer Tumore häufiger mit einer Ertaubung einhergeht, zeigen sehr kleine VS (≤1 cm) postoperative Hörerhaltungsraten zwischen 70–85 % [17]. Sowohl beim retrosigmoidealen als auch beim subtemporalen Zugang, welche beide primär gehörerhaltend sind, kann es naturgemäß zur Verschlechterung des funktionellen Hörens bis zur Ertaubung kommen. Dies gilt jedoch ebenso für die Langzeitergebnisse nach Bestrahlung als auch bei Observanz und kann selbst bei nicht wachsenden VS auftreten.

*Translabyrinthär*e VS Resektionen implizieren einen Zugang durch den vestibulären Anteil des Innenohrs und werden daher primär bei Patienten ohne funktionellem Gehör eingesetzt. Die hierbei durchgeführte Mastoidektomie und Labyrinthektomie ermöglicht die schonende Entfernung des VS, da auf das Kleinhirn (und den Temporallappen) kein Druck ausgeübt werden muss und der Tumor schonend exponiert wird. Die Möglichkeit der Durchführung einer simultanen Cochlea Implantation macht diesen Zugang besonders interessant.

**Figure 3:**
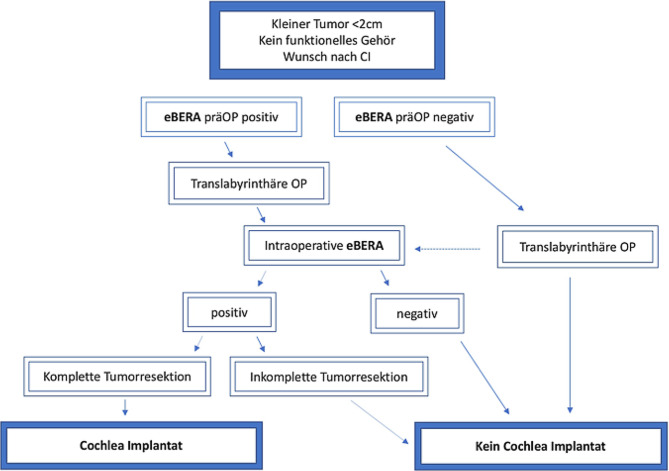

Wenige Zentren bieten alle drei chirurgischen Zugangswege und stereotaktische Bestrahlung als Therapiealternativen an. Als eine von wenigen Kliniken in Europa werden im Wiener AKH in enger Kooperation (Neurochirurgie und HNO) alle Therapiealternativen durchgeführt. Dies ermöglichte die chirurgische Resektion von insgesamt 63 VS alleine im Jahr 2019. Insbesondere im Bereich der translabyrinthären Resektion und simultanen Cochlea Implantation hat Wien eine Vorreiterrolle eingenommen und bereits erfreuliche Ergebnisse erzielt (Dahm et al. 2020, [18]). Dieser operative Zugang kann bei Patienten mit funktionslosem Gehör in Kombination mit VS kleiner als 2 cm angewendet werden. Die Funktion des Hörnervs wird hier mittels elektrisch evozierter Hirnstammaudiometrie (eBERA, [19]) prä- und intraoperativ ermittelt (Abb. 3). Ergibt die intraoperative Messung der eBERA ein positives Ergebnis, so kann eine Cochlea Implantation nach Tumorresektion durchgeführt werden. Bei negativer eBERA oder unvollständiger Tumorresektion wird auf eine Cochlea Implantation verzichtet. Die Vorteile für die Patienten sind evident: in einem einzigen Eingriff wird der Tumor entfernt und das Hörvermögen wiedererlangt. Dennoch wurde in der Vergangenheit dieses Prinzip nur in besonderen Fällen durchgeführt. Ein Umdenken hat vor allem aufgrund der MRT Tauglichkeit und Artefaktarmut der modernen Implantate, sowie aufgrund der intraoperativen Überwachung des Gesichts- und Hörnerven eingesetzt.

## Nachsorge

Nach der Operation werden Patienten üblicherweise eine Nacht auf der Intensivstation überwacht. Am ersten postoperativen Tag erfolgt ein CCT, welches primär zum Ausschluss einer intrakraniellen Blutung durchgeführt wird, bevor die Patienten auf die Normalstation verlegt werden können. Neben der Überprüfung der Fazialisfunktion und Wundkontrolle soll vor der Entlassung eine Tonaudiometrie durchgeführt werden. Abhängig von der präoperativen Hörfunktion und dem gewählten chirurgischen Zugang, sollten regelmäßige Hörtests im Sinne von Ton- und Sprachaudiometrien folgen. Während die ambulante Nachkontrolle in der Regel einige Wochen nach Entlassung des Patienten organisiert wird, gestaltet sich die weitere postoperative Nachsorge entsprechend des chirurgischen Resektionsausmaßes und kann je nach Therapiezentrum variieren.

Konnte das VS komplett reseziert werden, so wird die Durchführung eines Verlaufs-MRT ein Jahr nach Operation empfohlen [20]. Bei unauffälligem Befund sollte ein erneuter Scan im dritten Jahr erfolgen. Im Falle einer unvollständigen Resektion des VS ist ein zeitlich engeres Scan-Schema indiziert. Hierbei sollte jeweils eine MRT-Kontrolle drei bis sechs Monate und ein Jahr nach initialer Therapie erfolgen. Die nächsten Bildgebungs-Untersuchungen sollten in den Jahren zwei, drei und fünf erfolgen. Waren die durchgeführten Verlaufskontrollen unauffällig, so können sowohl nach Komplettresektion als auch nach erfolgter Teilresektion weitere MRT-Kontrollen beispielsweise zehn und 15 Jahre nach dem operativen Eingriff durchgeführt werden.

## Conclusio

Eine standardisierte Entscheidungsfindung für VS gibt es aufgrund der zahlreichen, Therapie beeinflussenden Faktoren, nicht. Das individuelle Management von VS ist komplex und bedarf der Kommunikation von interdisziplinären Experten dieses Gebiets. Obwohl es derzeit keine definierten Standards in der Therapie von VS gibt, sind Funktionserhalt und Lebensqualität die wichtigsten Parameter, an die heutzutage international mehr denn je Wert gelegt wird. Therapieentscheidungen können bei gleicher Symptomatik zweier Patienten unterschiedlich ausfallen. Aus diesem Grund sollten von unreflektierten Aussagen wie zum Beispiel „auf keinen Fall bestrahlen“ oder „diese Tumorgröße auf keinen Fall operieren“ Abstand gehalten werden. Eine Entscheidungsabnahme über die Therapie der Betroffenen sollte von Ärztinnen und Ärzten vermieden werden, auch wenn dies verständlicherweise von vielen Patienten verlangt wird. Stattdessen ist eine ausführliche Aufklärung des Patienten über das zeitliche Fenster der Therapiealternativen und der Verweis zu interdisziplinären Experten sinnvoll.
